# 4-(3-Fluoro­anilino)thieno[2,3-*b*]pyridine-6-carb­oxy­lic acid

**DOI:** 10.1107/S1600536812027195

**Published:** 2012-06-27

**Authors:** Luiz C. S. Pinheiro, Alice M. R. Bernardino, Solange M. S. V. Wardell, James L. Wardell, Edward R. T. Tiekink

**Affiliations:** a Fundação Oswaldo Cruz, Instituto de Tecnologia em Fármacos, Departamento de Síntese Orgânica, Manguinhos, CEP 21041-250, Rio de Janeiro, RJ, Brazil; b Universidade Federal Fluminense, Instituto de Química, Departamento de Química Orgânica, Programa de Pós-Graduação em Química Orgânica, Campus do Valonguinho, CEP 24210-150, Niterói, RJ, Brazil; c CHEMSOL, 1 Harcourt Road, Aberdeen AB15 5NY, Scotland; d Centro de Desenvolvimento Tecnológico em Saúde (CDTS), Fundação Oswaldo Cruz (FIOCRUZ), Casa Amarela, Campus de Manguinhos, Av. Brasil 4365, 21040-900 Rio de Janeiro, RJ, Brazil; e Department of Chemistry, University of Malaya, 50603 Kuala Lumpur, Malaysia

## Abstract

In the title compound, C_14_H_9_FN_2_O_2_S, the thieno[2,3-*b*]pyridine residue is almost planar (r.m.s. deviation = 0.0194 Å), with the carb­oxy­lic acid group [dihedral angle = 11.9 (3)°] and the benzene ring [71.1 (4)°] being twisted out of this plane to different extents. An intra­molecular N—H⋯O(carbon­yl) hydrogen bond closes an *S*(6) ring. Supra­molecular chains along [01-1] mediated by O—H⋯N(pyridine) hydrogen bonds feature in the crystal. A three-dimensional architecture is completed by π–π inter­actions occurring between the benzene ring and the two rings of the thieno[2,3-*b*]pyridine residue [centroid–centroid distances = 3.6963 (13) and 3.3812 (13) Å]. The F atom is disordered over the two *meta* sites in a near statistical ratio [0.545 (5):0.455 (5)].

## Related literature

For the biological activity of 4-(aryl­amino)­thieno[2,3-*b*]pyridine-5-carb­oxy­lic acids, see: Bernardino *et al.* (2007 ▶); Pinheiro *et al.* (2008 ▶). For the synthesis, see: Leal *et al.* (2008 ▶).
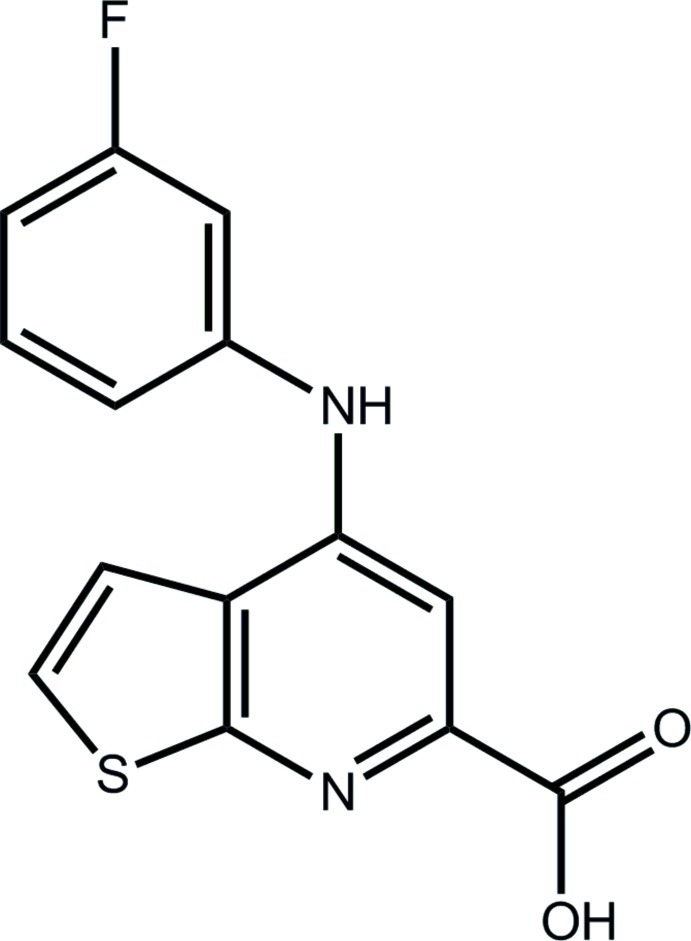


## Experimental

### 

#### Crystal data

C_14_H_9_FN_2_O_2_S
*M* *_r_* = 288.29
Orthorhombic, 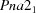

*a* = 17.0666 (6) Å
*b* = 8.7147 (3) Å
*c* = 7.9931 (2) Å
*V* = 1188.82 (7) Å^3^
*Z* = 4
Mo *K*α radiation
μ = 0.29 mm^−1^
*T* = 120 K
0.66 × 0.24 × 0.17 mm

#### Data collection

Bruker–Nonius Roper CCD camera on κ-goniostat diffractometer
Absorption correction: multi-scan (*SADABS*; Sheldrick, 2007 ▶) *T*_min_ = 0.788, *T*_max_ = 1.000
10259 measured reflections
2643 independent reflections
2376 reflections with *I* > 2σ(*I*)
*R*_int_ = 0.058

#### Refinement

*R*[*F* ^2^ > 2σ(*F* ^2^)] = 0.035
*wR*(*F* ^2^) = 0.087
*S* = 1.06
2643 reflections
197 parameters
3 restraints
H atoms treated by a mixture of independent and constrained refinement
Δρ_max_ = 0.20 e Å^−3^
Δρ_min_ = −0.42 e Å^−3^
Absolute structure: Flack (1983 ▶), 1181 Friedel pairs
Flack parameter: 0.15 (9)

### 

Data collection: *COLLECT* (Hooft, 1998 ▶); cell refinement: *DENZO* (Otwinowski & Minor, 1997 ▶) and *COLLECT*; data reduction: *DENZO* and *COLLECT*; program(s) used to solve structure: *SHELXS97* (Sheldrick, 2008 ▶); program(s) used to refine structure: *SHELXL97* (Sheldrick, 2008 ▶); molecular graphics: *ORTEP-3* (Farrugia, 1997 ▶) and *DIAMOND* (Brandenburg, 2006 ▶); software used to prepare material for publication: *publCIF* (Westrip, 2010 ▶).

## Supplementary Material

Crystal structure: contains datablock(s) global, I. DOI: 10.1107/S1600536812027195/hb6844sup1.cif

Structure factors: contains datablock(s) I. DOI: 10.1107/S1600536812027195/hb6844Isup2.hkl

Supplementary material file. DOI: 10.1107/S1600536812027195/hb6844Isup3.cml

Additional supplementary materials:  crystallographic information; 3D view; checkCIF report

## Figures and Tables

**Table 1:** Hydrogen-bond geometry (Å, °)

*D*—H⋯*A*	*D*—H	H⋯*A*	*D*⋯*A*	*D*—H⋯*A*
O1—H1o⋯N1^i^	0.86 (2)	1.84 (2)	2.681 (2)	167 (3)
N2—H2n⋯O2	0.88 (2)	1.85 (2)	2.635 (2)	147 (2)

Symmetry code: (i) 
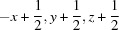
.

**Fig. 1.**
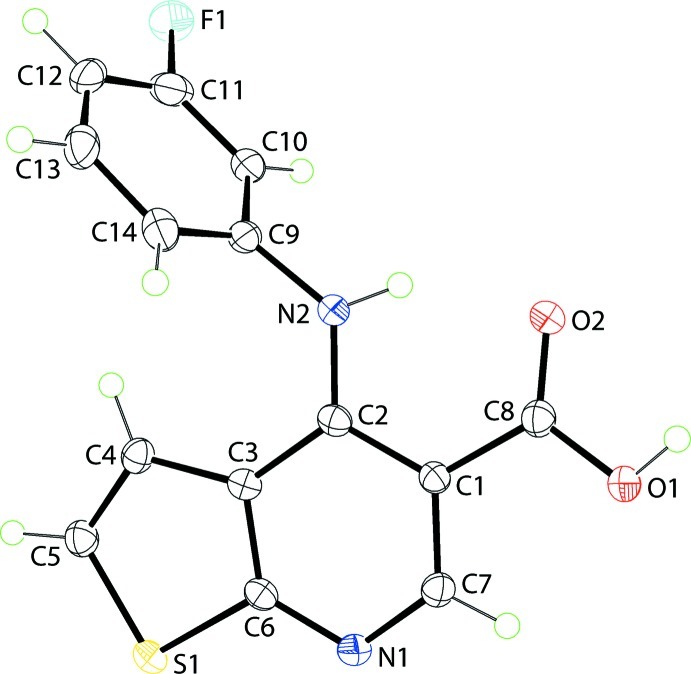
The molecular structure of (I) showing displacement ellipsoids at the 50% probability level.

**Fig. 2.**
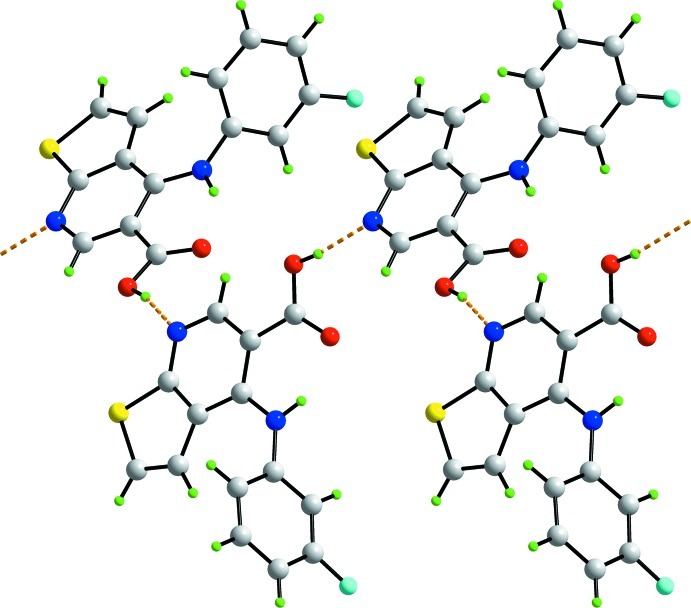
A view of the supramolecular chain in (I) sustained by O—H···N (orange dashed lines) hydrogen bonds.

**Fig. 3.**
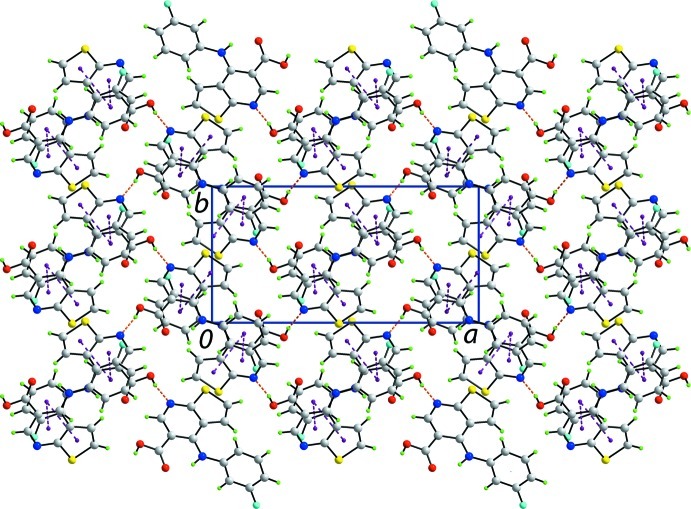
A view in projection down the c axis of the unit-cell contents for (I). The O—H···N and π—π interactions are shown as orange and purple dashed lines, respectively.

## supplementary crystallographic information

### Comment

Among the reported thienopyridine derivatives is a family of 4-(arylamino)thieno[2,3-*b*]pyridine-5-carboxylic acids, whose anti-virial and anti-bacterial activities have been investigated (Leal *et al.*, 2008; Bernardino *et al.*, 2007; Pinheiro *et al.*, 2008). The structure of one of these derivatives, the title compound (I), is now reported.

In (I), Fig. 1, the nine atoms comprising the thieno[2,3-*b*]pyridine fused ring system are planar with a r.m.s. deviation of 0.0194 Å and maximum deviations of 0.0343 (19) and -0.0227 (16) for the C7 and C6 atoms, respectively. The carboxylic acid residue is twisted out of this plane, forming a dihedral angle of 11.9 (3)°, and the terminal benzene ring, which is orientated towards the thienyl ring, is almost orthogonal, the dihedral angle being 71.1 (4)°. There is an intramolecular *N*—*H*···*O*(carbonyl) hydrogen bond, Table 1, which closes an *S*(6) loop.

In the crystal packing, supramolecular chains along [0 1 - 1] are formed *via* O—H···N(pyridyl) hydrogen bonds, Fig. 2 and Table 1. These are consolidated into a three-dimensional architecture *via*π—π interactions whereby the thieno[2,3-*b*]pyridine residue is straddled by a symmetry related benzene ring [inter-centroid (thienyl···benzene) distance = 3.6963 (13) Å, angle of inclination = 1.41 (12)°, and inter-centroid (pyridyl···benzene) distance = 3.3812 (13) Å and angle of inclination = 3.25 (11)° for symmetry operation -*x*, 2 - *y*, -1/2 + *z*], Fig. 3.

### Experimental

The title compound was prepared as reported (Leal *et al.*, 2008). The dark orange blade used in the structure determination was grown from its toluene/acetonitrile solution.

### Refinement

The C-bound H atoms were geometrically placed (C—H = 0.95 Å) and refined as riding with *U*_iso_(H) = 1.2*U*_eq_(C). The O– and N-bound H atoms were located from a difference map and refined with distance restraints of O—H = 0.84±0.01 and N—H = 0.88±0.01 Å, and with *U*_iso_(H) = *zU*_eq_(carrier atom); *z* = 1.5 for O and *z* = 1.2 for N. The F1 atom is disordered over two position. Each site was refined with individual anisotropic displacement parameters. The major component refined to a site occupancy factor = 0.545 (5). The (022) reflection was omitted from the final refinement owing to poor agreement.

### Crystal data

**Table:**

C_14_H_9_FN_2_O_2_S	*F*(000) = 592
*M**_r_* = 288.29	*D*_x_ = 1.611 Mg m^−^^3^
Orthorhombic, *P**n**a*2_1_	Mo *K*α radiation, λ = 0.71073 Å
Hall symbol: P 2c -2n	Cell parameters from 1601 reflections
*a* = 17.0666 (6) Å	θ = 1.0–27.5°
*b* = 8.7147 (3) Å	µ = 0.29 mm^−^^1^
*c* = 7.9931 (2) Å	*T* = 120 K
*V* = 1188.82 (7) Å^3^	Blade, dark-orange
*Z* = 4	0.66 × 0.24 × 0.17 mm

### Data collection

**Table:**

Bruker–Nonius Roper CCD camera on κ-goniostat diffractometer	2643 independent reflections
Radiation source: Bruker-Nonius FR591 rotating anode	2376 reflections with *I* > 2σ(*I*)
Graphite monochromator	*R*_int_ = 0.058
Detector resolution: 9.091 pixels mm^-1^	θ_max_ = 27.5°, θ_min_ = 3.5°
φ & ω scans	*h* = −22→22
Absorption correction: multi-scan (*SADABS*; Sheldrick, 2007)	*k* = −11→11
*T*_min_ = 0.788, *T*_max_ = 1.000	*l* = −10→9
10259 measured reflections	

### Refinement

**Table:**

Refinement on *F*^2^	Secondary atom site location: difference Fourier map
Least-squares matrix: full	Hydrogen site location: inferred from neighbouring sites
*R*[*F*^2^ > 2σ(*F*^2^)] = 0.035	H atoms treated by a mixture of independent and constrained refinement
*wR*(*F*^2^) = 0.087	*w* = 1/[σ^2^(*F*_o_^2^) + (0.0375*P*)^2^ + 0.2847*P*] where *P* = (*F*_o_^2^ + 2*F*_c_^2^)/3
*S* = 1.06	(Δ/σ)_max_ < 0.001
2643 reflections	Δρ_max_ = 0.20 e Å^−^^3^
197 parameters	Δρ_min_ = −0.42 e Å^−^^3^
3 restraints	Absolute structure: Flack (1983), 1181 Friedel pairs
Primary atom site location: structure-invariant direct methods	Flack parameter: 0.15 (9)

### Special details

**Table:**

Geometry. All s.u.'s (except the s.u. in the dihedral angle between two l.s. planes) are estimated using the full covariance matrix. The cell s.u.'s are taken into account individually in the estimation of s.u.'s in distances, angles and torsion angles; correlations between s.u.'s in cell parameters are only used when they are defined by crystal symmetry. An approximate (isotropic) treatment of cell s.u.'s is used for estimating s.u.'s involving l.s. planes.
Refinement. Refinement of *F*^2^ against ALL reflections. The weighted *R*-factor *wR* and goodness of fit *S* are based on *F*^2^, conventional *R*-factors *R* are based on *F*, with *F* set to zero for negative *F*^2^. The threshold expression of *F*^2^ > 2σ(*F*^2^) is used only for calculating *R*-factors(gt) *etc*. and is not relevant to the choice of reflections for refinement. *R*-factors based on *F*^2^ are statistically about twice as large as those based on *F*, and *R*- factors based on ALL data will be even larger.

### Fractional atomic coordinates and isotropic or equivalent isotropic displacement parameters (Å^2^)

**Table:**

	*x*	*y*	*z*	*U*_iso_*/*U*_eq_	Occ. (<1)
S1	0.02466 (3)	0.49277 (6)	0.56709 (9)	0.02149 (14)	
O1	0.26956 (9)	0.89190 (18)	1.0209 (2)	0.0246 (4)	
H1O	0.2865 (16)	0.959 (2)	1.090 (3)	0.037*	
O2	0.17443 (8)	1.06688 (18)	1.0208 (2)	0.0254 (4)	
N1	0.15569 (10)	0.5951 (2)	0.7157 (2)	0.0185 (4)	
N2	0.03616 (11)	0.9821 (2)	0.9068 (3)	0.0214 (4)	
H2N	0.0711 (11)	1.041 (3)	0.956 (3)	0.026*	
C1	0.18990 (13)	0.7023 (2)	0.8094 (3)	0.0184 (5)	
H1	0.2445	0.6918	0.8298	0.022*	
C2	0.15232 (12)	0.8288 (2)	0.8800 (2)	0.0161 (4)	
C3	0.07089 (13)	0.8526 (2)	0.8490 (3)	0.0167 (4)	
C4	0.03239 (12)	0.7380 (2)	0.7525 (3)	0.0154 (4)	
C5	−0.04725 (13)	0.7259 (2)	0.6927 (3)	0.0190 (5)	
H5	−0.0872	0.7977	0.7195	0.023*	
C6	−0.05889 (12)	0.6029 (2)	0.5952 (3)	0.0214 (5)	
H6	−0.1081	0.5784	0.5464	0.026*	
C7	0.07779 (12)	0.6170 (2)	0.6923 (3)	0.0172 (4)	
C8	0.19891 (12)	0.9401 (3)	0.9793 (3)	0.0198 (5)	
C9	−0.04436 (13)	1.0256 (3)	0.9067 (3)	0.0197 (5)	
C10	−0.06524 (14)	1.1599 (3)	0.8256 (3)	0.0222 (5)	
H10	−0.0279	1.2153	0.7613	0.027*	
C12	−0.19667 (14)	1.1360 (3)	0.9319 (4)	0.0341 (6)	
H12	−0.2487	1.1741	0.9406	0.041*	
C14	−0.09947 (13)	0.9457 (3)	1.0002 (3)	0.0245 (5)	
H14	−0.0857	0.8531	1.0554	0.029*	
C11	−0.14136 (15)	1.2115 (3)	0.8400 (3)	0.0315 (6)	0.545 (5)
C13	−0.17454 (14)	1.0032 (3)	1.0113 (3)	0.0310 (6)	0.545 (5)
H13	−0.2122	0.9491	1.0761	0.037*	0.545 (5)
F1	−0.16627 (15)	1.3395 (3)	0.7761 (3)	0.0330 (9)	0.545 (5)
C11'	−0.14136 (15)	1.2115 (3)	0.8400 (3)	0.0315 (6)	0.455 (5)
H11	−0.1559	1.3032	0.7838	0.038*	0.455 (5)
C13'	−0.17454 (14)	1.0032 (3)	1.0113 (3)	0.0310 (6)	0.455 (5)
F1'	−0.23113 (18)	0.9534 (4)	1.1023 (4)	0.0392 (12)	0.455 (5)

### Atomic displacement parameters (Å^2^)

**Table:**

	*U*^11^	*U*^22^	*U*^33^	*U*^12^	*U*^13^	*U*^23^
S1	0.0221 (2)	0.0178 (2)	0.0245 (3)	−0.0018 (2)	−0.0014 (3)	−0.0059 (2)
O1	0.0187 (7)	0.0219 (8)	0.0332 (9)	0.0019 (6)	−0.0077 (6)	−0.0077 (7)
O2	0.0198 (8)	0.0194 (8)	0.0370 (10)	−0.0001 (6)	−0.0014 (7)	−0.0092 (7)
N1	0.0181 (9)	0.0169 (9)	0.0205 (9)	0.0010 (7)	0.0011 (7)	−0.0018 (8)
N2	0.0165 (9)	0.0178 (9)	0.0300 (11)	0.0007 (7)	−0.0026 (8)	−0.0070 (8)
C1	0.0175 (10)	0.0170 (10)	0.0208 (12)	0.0006 (8)	−0.0013 (8)	0.0037 (9)
C2	0.0162 (10)	0.0149 (9)	0.0171 (11)	−0.0008 (8)	−0.0004 (8)	−0.0001 (8)
C3	0.0198 (11)	0.0139 (10)	0.0163 (10)	−0.0010 (8)	0.0016 (8)	0.0019 (9)
C4	0.0194 (10)	0.0147 (10)	0.0123 (10)	0.0002 (8)	0.0006 (8)	0.0015 (8)
C5	0.0172 (10)	0.0201 (11)	0.0197 (11)	0.0003 (8)	−0.0007 (8)	0.0030 (9)
C6	0.0203 (10)	0.0213 (10)	0.0226 (12)	−0.0011 (8)	−0.0030 (9)	−0.0002 (9)
C7	0.0204 (10)	0.0145 (10)	0.0165 (10)	−0.0013 (8)	0.0003 (8)	0.0006 (8)
C8	0.0184 (11)	0.0211 (11)	0.0199 (12)	−0.0006 (9)	−0.0015 (9)	−0.0005 (9)
C9	0.0178 (10)	0.0219 (11)	0.0194 (11)	0.0016 (9)	−0.0033 (8)	−0.0071 (9)
C10	0.0214 (12)	0.0197 (11)	0.0256 (12)	−0.0001 (10)	−0.0029 (9)	−0.0040 (10)
C12	0.0170 (11)	0.0419 (15)	0.0434 (15)	0.0045 (11)	−0.0050 (11)	−0.0252 (13)
C14	0.0249 (12)	0.0257 (11)	0.0230 (12)	−0.0059 (10)	0.0003 (10)	−0.0021 (10)
C11	0.0325 (14)	0.0242 (12)	0.0379 (15)	0.0084 (11)	−0.0111 (12)	−0.0113 (11)
C13	0.0208 (12)	0.0425 (16)	0.0296 (14)	−0.0062 (10)	0.0022 (10)	−0.0162 (12)
F1	0.0327 (16)	0.0212 (14)	0.0452 (18)	0.0071 (12)	−0.0064 (12)	0.0067 (12)
C11'	0.0325 (14)	0.0242 (12)	0.0379 (15)	0.0084 (11)	−0.0111 (12)	−0.0113 (11)
C13'	0.0208 (12)	0.0425 (16)	0.0296 (14)	−0.0062 (10)	0.0022 (10)	−0.0162 (12)
F1'	0.0281 (18)	0.046 (2)	0.044 (2)	−0.0163 (14)	0.0099 (15)	−0.0147 (18)

### Geometric parameters (Å, º)

**Table:**

S1—C7	1.731 (2)	C6—H6	0.9500
S1—C6	1.733 (2)	C9—C14	1.388 (3)
O1—C8	1.319 (3)	C9—C10	1.384 (3)
O1—H1O	0.852 (10)	C10—C11'	1.380 (3)
O2—C8	1.227 (3)	C10—C11	1.380 (3)
N1—C1	1.332 (3)	C10—H10	0.9500
N1—C7	1.356 (3)	C12—C11'	1.365 (4)
N2—C3	1.356 (3)	C12—C11	1.365 (4)
N2—C9	1.426 (3)	C12—C13'	1.373 (4)
N2—H2N	0.881 (10)	C12—C13	1.373 (4)
C1—C2	1.394 (3)	C12—H12	0.9500
C1—H1	0.9500	C14—C13'	1.378 (3)
C2—C3	1.427 (3)	C14—C13	1.378 (3)
C2—C8	1.484 (3)	C14—H14	0.9500
C3—C4	1.422 (3)	C11—F1	1.298 (4)
C4—C7	1.394 (3)	C13—H13	0.9500
C4—C5	1.445 (3)	C11'—H11	0.9500
C5—C6	1.340 (3)	C13'—F1'	1.284 (4)
C5—H5	0.9500		
			
C7—S1—C6	90.55 (10)	C11'—C10—C9	118.6 (2)
C8—O1—H1O	104.9 (19)	C11—C10—C9	118.6 (2)
C1—N1—C7	114.12 (18)	C11'—C10—H10	120.7
C3—N2—C9	129.99 (18)	C11—C10—H10	120.7
C3—N2—H2N	110.0 (17)	C9—C10—H10	120.7
C9—N2—H2N	119.9 (17)	C11'—C12—C11	0.0 (3)
N1—C1—C2	125.5 (2)	C11'—C12—C13'	117.7 (2)
N1—C1—H1	117.3	C11—C12—C13'	117.7 (2)
C2—C1—H1	117.3	C11'—C12—C13	117.7 (2)
C1—C2—C3	119.5 (2)	C11—C12—C13	117.7 (2)
C1—C2—C8	119.12 (19)	C13'—C12—C13	0.0 (2)
C3—C2—C8	121.30 (19)	C11'—C12—H12	121.1
N2—C3—C4	124.5 (2)	C11—C12—H12	121.1
N2—C3—C2	119.2 (2)	C13'—C12—H12	121.1
C4—C3—C2	116.25 (18)	C13—C12—H12	121.1
C7—C4—C3	117.46 (18)	C9—C14—C13'	118.9 (2)
C7—C4—C5	110.70 (19)	C9—C14—C13	118.9 (2)
C3—C4—C5	131.71 (19)	C13'—C14—C13	0.00 (7)
C6—C5—C4	113.0 (2)	C9—C14—H14	120.6
C6—C5—H5	123.5	C13'—C14—H14	120.6
C4—C5—H5	123.5	C13—C14—H14	120.6
C5—C6—S1	113.36 (17)	F1—C11—C12	113.5 (3)
C5—C6—H6	123.3	F1—C11—C10	123.8 (3)
S1—C6—H6	123.3	C12—C11—C10	122.6 (2)
N1—C7—C4	127.12 (19)	C12—C13—C14	122.1 (2)
N1—C7—S1	120.36 (16)	C12—C13—H13	118.9
C4—C7—S1	112.43 (15)	C14—C13—H13	118.9
O2—C8—O1	122.0 (2)	C12—C11'—C10	122.6 (2)
O2—C8—C2	123.44 (19)	C12—C11'—H11	118.7
O1—C8—C2	114.57 (19)	C10—C11'—H11	118.7
C14—C9—C10	120.1 (2)	F1'—C13'—C12	109.9 (3)
C14—C9—N2	121.3 (2)	F1'—C13'—C14	127.8 (3)
C10—C9—N2	118.2 (2)	C12—C13'—C14	122.1 (2)
C11'—C10—C11	0.0 (2)		
			
C7—N1—C1—C2	−0.3 (3)	C10—C9—C14—C13'	−0.5 (3)
N1—C1—C2—C3	−1.9 (3)	N2—C9—C14—C13'	172.4 (2)
N1—C1—C2—C8	−178.91 (19)	C10—C9—C14—C13	−0.5 (3)
C9—N2—C3—C4	9.1 (4)	N2—C9—C14—C13	172.4 (2)
C9—N2—C3—C2	−173.2 (2)	C11'—C12—C11—F1	0 (97)
C1—C2—C3—N2	−174.7 (2)	C13'—C12—C11—F1	−176.8 (2)
C8—C2—C3—N2	2.2 (3)	C13—C12—C11—F1	−176.8 (2)
C1—C2—C3—C4	3.1 (3)	C11'—C12—C11—C10	0 (100)
C8—C2—C3—C4	−179.95 (19)	C13'—C12—C11—C10	−0.4 (4)
N2—C3—C4—C7	175.4 (2)	C13—C12—C11—C10	−0.4 (4)
C2—C3—C4—C7	−2.3 (3)	C11'—C10—C11—F1	0 (100)
N2—C3—C4—C5	0.0 (4)	C9—C10—C11—F1	176.5 (3)
C2—C3—C4—C5	−177.7 (2)	C11'—C10—C11—C12	0 (75)
C7—C4—C5—C6	0.2 (3)	C9—C10—C11—C12	0.4 (4)
C3—C4—C5—C6	175.9 (2)	C11'—C12—C13—C14	−0.1 (4)
C4—C5—C6—S1	−0.3 (3)	C11—C12—C13—C14	−0.1 (4)
C7—S1—C6—C5	0.20 (18)	C13'—C12—C13—C14	0 (100)
C1—N1—C7—C4	1.2 (3)	C9—C14—C13—C12	0.6 (4)
C1—N1—C7—S1	177.51 (16)	C13'—C14—C13—C12	0 (100)
C3—C4—C7—N1	0.2 (3)	C11—C12—C11'—C10	0 (100)
C5—C4—C7—N1	176.5 (2)	C13'—C12—C11'—C10	−0.4 (4)
C3—C4—C7—S1	−176.41 (16)	C13—C12—C11'—C10	−0.4 (4)
C5—C4—C7—S1	−0.1 (2)	C11—C10—C11'—C12	0 (75)
C6—S1—C7—N1	−176.90 (18)	C9—C10—C11'—C12	0.4 (4)
C6—S1—C7—C4	−0.07 (17)	C11'—C12—C13'—F1'	174.9 (3)
C1—C2—C8—O2	166.8 (2)	C11—C12—C13'—F1'	174.9 (3)
C3—C2—C8—O2	−10.2 (3)	C13—C12—C13'—F1'	0 (59)
C1—C2—C8—O1	−13.5 (3)	C11'—C12—C13'—C14	−0.1 (4)
C3—C2—C8—O1	169.52 (19)	C11—C12—C13'—C14	−0.1 (4)
C3—N2—C9—C14	65.4 (4)	C13—C12—C13'—C14	0 (100)
C3—N2—C9—C10	−121.5 (3)	C9—C14—C13'—F1'	−173.6 (3)
C14—C9—C10—C11'	0.0 (3)	C13—C14—C13'—F1'	0 (79)
N2—C9—C10—C11'	−173.1 (2)	C9—C14—C13'—C12	0.6 (4)
C14—C9—C10—C11	0.0 (3)	C13—C14—C13'—C12	0 (100)
N2—C9—C10—C11	−173.1 (2)		

### Hydrogen-bond geometry (Å, º)

**Table:**

*D*—H···*A*	*D*—H	H···*A*	*D*···*A*	*D*—H···*A*
O1—H1o···N1^i^	0.86 (2)	1.84 (2)	2.681 (2)	167 (3)
N2—H2n···O2	0.88 (2)	1.85 (2)	2.635 (2)	147 (2)

Symmetry code: (i) −*x*+1/2, *y*+1/2, *z*+1/2.
